# Unexpected ocular morphological changes after corneal refractive surgery: A review

**DOI:** 10.3389/fmed.2022.1014277

**Published:** 2022-11-29

**Authors:** Maddalena De Bernardo, Sergio Pagliarulo, Nicola Rosa

**Affiliations:** Eye Unit, Department of Medicine Surgery and Dentistry, “Scuola Medica Salernitana”, University of Salerno, Salerno, Italy

**Keywords:** corneal refractive surgery, anterior chamber depth, axial length, RK, PRK, LASEK, LASIK

## Abstract

Corneal refractive surgery (CRS) currently is widely used to correct refractive errors because of its efficacy and reliability. Several studies dealt with the corneal modification induced by this type of surgery, but it is still debated if CRS can induce unexpected changes namely anterior chamber depth (ACD) and axial length (AL). A literature review was performed, including all articles regarding CRS and eye-variations from 1999 to December 2021. Excluding articles about specific systemic conditions (e.g., pregnancy), pathological conditions, post-surgical complications or about only corneal flattening and thinning post CRS, we found nine studies that met the search criteria. We divided the found articles according to the type of surgery performed (radial keratotomy, PRK/LASEK, lasik) and analyzed the results about ACD and AL. Finally, according to the literature, we can conclude that CRS not only gives a corneal flattening, thinning and biomechanical changes, but also induces AL and ACD decrease. This makes the AL and ACD measurements obtained before CRS uselessness in case of IOL power calculation.

## Introduction

Since the first studies performed in the 80's of last century, corneal refractive surgery (CRS) has been adopted as a leading method to correct refractive errors (1, 2). Thanks to its reliability and effectiveness, today it is considered a worldwide diffuse and accepted method (3–7). It is well-known that the refractive effect is related to the corneal flattening and thinning, and several authors evaluated these induced changes (8–11).

CRS not only changes the corneal shape, but also its biomechanical properties increasing the risk to develop ectasia, mainly in some specifical cases (12, 13).

It is also recognized that these changes make the intraocular pressure (14, 15) and corneal power measurements unreliable (16–19), troubling the calculation of the intraocular lens power to be implanted in case of cataract surgery (20, 21).

On the other hand, as the cornea is not a piece of plastic so “any procedure that circumferentially severs lamellae will flatten the cornea centrally due to an outward force in the periphery pulling laterally on the center, will flatten the cornea centrally” (22), it should be obvious that the induced corneal changes could give a corneal weakening with successive changes in the corneal curvature, and consequently changes in the anterior chamber depth (ACD) and/or axial length (AL).

The purpose of this paper was to review the data available on this topic and to discuss the possible reasons.

## Materials and methods

Literature search was performed utilizing the PubMed medical database. The database search approach was developed using several keywords and text words, referred to CRS and eye changes such as corneal changes (excluding paper where just corneal thickness and flattening were evaluated), anterior chamber depth, axial length, corneal remodeling, lens thickness. Articles involving specific systemic conditions (e.g., pregnancy), pathological conditions or post-surgical complications were also excluded.

Based on the above methods, the earliest useful publication was in 1999, and our research ended in December 2021. There were no language limits on searches, but only English-language publications were evaluated. For further inclusions, the initial search's references were carefully checked.

## Results

The search found 28 articles and 9 of them were retained as relevant articles (Table 1). In particular: one paper was related to changes occurred after Radial Keratotomy (RK) (23), six papers after Photorefractive Keratectomy/Laser-Assisted Sub-Epithelial Keratectomy (PRK/LASEK) (7, 24–28), and two papers after Laser-Assisted *in situ* Keratomileusis (LASIK) (29, 30).

**Table 1:** Methods and results of the studies (significant values are in bold type).

**References**	**Methods**	**Al pre mean ±SD (mm)**	**Al post mean ±SD (mm)**	**Acd pre mean ±SD (mm)**	**Acd post mean ±SD (mm)**	**Differences in al (pre—post) mean ±SD (mm)**	**Differences in acd (pre—post) mean ±SD (mm)**	**Corneal ablation depth (micron)**
Demirok et al. (23)	RK group 1	Preoperative 24.56 ± 0.74	3rd day 24.37 ± 0.77 2nd week 24.48 ± 0.75 3rd month 24.49 ± 0.74 6th month 24.47 ± 0.74	Preoperative 3.60 ± 0.25	3rd day 3.39 ± 0.26 2nd week 3.51 ± 0.25 3rd month 3.48 ± 0.22 6th month 3.51 ± 0.23	0.19 *p* = 0.36 0.08 *p* = 0.32 0.07 *p* = 0.69 0.09 *p* = 0.64	**0.21** ***p*** **=** **0.003** 0.09 *p* = 0.18 0.12 *p* = 0.06 0.09 *p* = 0.18	
	RK group 2	Preoperative 25.64 ± 1.04	3rd day 25.43 ± 1.06 2nd week 25.49 ± 1.05 3rd month 25.51 ± 1.03 6th month 25.52 ± 1.07	Preoperative 3.72 ± 0.26	3rd day 3.46 ± 0.26 2nd week 3.51 ± 0.25 3rd month 3.58 ± 0.27 6th month 3.59 ± 0.33	0.21 *p* = 0.47 0.15 *p* = 0.66 0.13 *p* = 0.65 0.12 *p* = 0.67	**0.26** ***p*** **<** **0.001 0.21** ***p*** **=** **0.007 0.14** ***p*** **=** **0.041** 0.13 *p* = 0.15	
Winkler von Mohrenfels et al. (24)	LASEK	Preoperative 25.46 ± 1.03	1st month 25.38 ± 0.99			0.08 *r* = 0.998		
Rosa et al. (25)	PRK	Preoperative 25.61 ± 1.47	1st month 25.48 ± 1.43			**0.13** **±0.07** ***p*** **<** **0.001**		(theoretical) 103.07 ± 45.06
	PRK	1st month 25.48 ± 1.43	3rd month 25.61 ± 1.36			−0.01 ± 0.05 *p* = 0.014		
	PRK	3rd month 25.61 ± 1.36	6th month 25.58 ± 1.35			0.01 ± 0.05 *p* = 0.24		
Rosa et al. (26)	PRK—Orbscan II (epithelium)			Preoperative 3.66 ± 0.34	1st month 3.54 ± 0.33		**0.12** ***p** **<*** **0.01**	
	PRK—Orbscan II (epithelium)			3rd month 3.57 ± 0.31	6th month 3.53 ± 0.34		0.04 *p* > 0.01	
	PRK—Orbscan II (endothelium)			Preoperative 3.07 ± 0.35	1st month 3.08 ± 0.35		0.01 *p* > 0.01	
	PRK—Orbscan II (endothelium)			3rd month 3.1 ± 0.33	6th month 3.06 ± 0.35		**0.04** ***p*** **<** **0.01**	
	PRK–IOLMaster			Preoperative 3.62 ± 0.33	1st month 3.5 ± 0.32		**0.12** ***p*** **<** **0.01**	
	PRK–IOLMaster			3rd month 3.52 ± 0.29	6th month 3.52 ± 0.31		0.00 *p* > 0.01	
Rosa et al. (27)	PRK	Preoperative 21.99 ± 1.09	1st month 25.48 ± 1.43			0.02 ± 0.03 *p* = 0.96		
		Preoperative 21.99 ± 1.09	3rd month 25.61 ± 1.36			0.03 ± 0.03 *p* = 0.99		
		Preoperative 21.99 ± 1.09	6th month 25.58 ± 1.35			0.02 ± 0.04 *p* = 0.31		
O'Brart et al. (7)	PRK	Preoperative 25.02 ± 0.91	20th year 25.86 ± 1.03	Preoperative 3.6 ± 0.38	20th year 3.19 ± 0.59	–**0.84** **±0.43** ***p*** **<** **0.0001**	**0.42** **±0.68** ***p*** **<** **0.02**	
De Bernardo et al. (28)	PRK -pentacam endothelium			Preoperative	1st month		**0.03** **±0.07** ***p*** **<** **0.05**	
	PRK -pentacam endothelium			Preoperative	3rd month		**0.04** **±0.08** ***p*** **<** **0.05**	
	PRK -pentacam endothelium			Preoperative	6th month		**0.04** **±0.09** ***p*** **<** **0.05**	
	PRK -pentacam epithelium			Preoperative	1st month		**0.13** **±0.09** ***p*** **<** **0.05**	
	PRK -pentacam epithelium			Preoperative	3rd month		**0.12** **±0.09** ***p*** **<** **0.05**	
	PRK -pentacam epithelium			Preoperative	6th month		**0.12** **±0.09** ***p*** **<** **0.05**	
	PRK—IOLMaster			Preoperative	1st month		**0.09** **±0.15** ***p*** **<** **0.05**	
	PRK–IOLMaster			Preoperative	3rd month		**0.14** **±0.16** ***p*** **<** **0.05**	
	PRK–IOLMaster			Preoperative	6th month		**0.17** **±0.16** ***p*** **<** **0.05**	
Chalkiadakis et al. (29)	LASIK	Preoperative 25.80 ± 1.01	1st month 25.68 ± 0.93			**0.12** **±0.10** ***p*** **<** **0.05**		(Theoretical) 73.4 ± 16.7
Wang et al. (30)	LASIK—IOLMaster	Preoperative 25.76 ± 1.02	1st week 25.75 ± 1.02	Preoperative 3.25 ± 0.25	1st week 3.21 ± 0.24	0.12 *p* > 0.05	**0.04** ***p*** **=** **0.009**	
		Preoperative 25.76 ± 1.02	1st month 25.76 ± 1.02	Preoperative 3.25 ± 0.25	1st month 3.21 ± 0.24	0.00 *p* > 0.05	**0.034** ***p*** **=** **0.002**	
		Preoperative 25.76 ± 1.02	3rd month 25.76 ± 1.03	Preoperative 3.25 ± 0.25	3rd month 3.21 ± 0.24	0.00 *p* > 0.05	**0.04** ***p*** **=** **0.001**	
		Preoperative 25.76 ± 1.02	6th month 25.76 ± 1.02	Preoperative 3.25 ± 0.25	6th month 3.21 ± 0.24	0.05 *p* > 0.05	**0.036** ***p*** **=** **0.003**	

### Changes after RK

In 1999 Demirok et al. (23) analyzed ACD and AL changes after Radial Keratotomy (RK) in 112 eyes of 58 myopic patients clustered into two groups: 70 eyes with a myopia correction <4.00 D (GROUP 1), and 42 eyes with a myopia correction >4.00 D (GROUP 2).

Authors assessed ACD and AL pre-operatively and on the third day, second week, third month and sixth month post-operatively.

GROUP 1: ACD in all measurements decreased post-operatively, but a statistically significant difference was observed on the third day only. AL decrease, without statistically significant differences, at all post-operative measurements was detected.

In GROUP 2, both ACD and AL decreased at all postoperative measurement times, with statistically significant change for ACD only at 3 days, 2 weeks, and 3 months post-operatively, that became not statistically significant at 6 months.

### Changes after PRK/LASEK

In 2003 Winkler von Mohrenfels et al. (24) using the IOLMaster, measured AL in 20 eyes with a myopia ranging from −2.75 to −8.00 diopters before and 1 month after Laser-Assisted Sub-Epithelial Keratectomy (LASEK). The authors found a good correlation between the values interpreting this as a non-significant difference concluded that results showed a not significant difference at follow up measurement.

In 2005 Rosa et al. (25), utilizing an IOL Master, measured the AL before and after Myopic Photorefractive Keratectomy (PRK) in 184 eyes of 133 patients, finding a decrease in the AL. Moreover, they tried to correlate the AL changes with theoretical ablation depth and refractive changes.

Through a linear regression analysis, they found a poor correlation between AL changes and the theoretical ablation depth (*R*^2^ = 0.3939) with a difference ranging from −82.95 to 133.66 μm (mean: 25.35 ± 54.15 μm) (*p* < 0.001) and with a tendency to increase with the increasing of refractive treatment. The AL difference between 1- and 3-months follow up was statistically significant.

The refractive modification and the AL difference between 1 and 3 months showed a poor correlation (*R*^2^ = 0.0084). In patients with 6-month follow-up, no statistically significant (*p* = 0.24) differences were found with the 3 months values. Correlation between the refractive changes and the AL difference between 3 and 6 months was poor (*R*^2^ = 0.0014).

In 2006 Rosa et al. (26) in another study, compared ACD measurements before and after PRK performed for myopia, hyperopia, and hyperopic, myopic, or mixed astigmatism [from −13.13 diopters (D) to +7 D; mean −3.67 ± 3.58] on 143 eyes of 143 patients. ACD measurements were obtained preoperatively and at one, three, and 6 months postoperatively with two different devices, IOLMaster and Orbscan II.

Measuring ACD from the epithelium, both devices detected a significant reduction between the preoperative and 1-month postoperative measurements, however a non-significant ACD reduction was showed between 3 and 6 months after treatment. In the ACD assessed from the endothelium with Orbscan II, only between the 3- and 6-month follow up, a statistically significant difference was found.

In 2013 Rosa et al. (27) evaluated the AL measurements before and after Hyperopic PRK in 61 eyes of 37 patients [refractive errors from +0.25 to +7 diopters (D); mean: +3.81 ± 1.62 D]. Authors used IOLMaster for AL measurements, and Oculus Pentacam to assess the central corneal thickness (CCT). Data were taken preoperatively and at 1, 3, and 6 months of follow-up after excimer laser treatment. After hyperopic PRK, neither the axial length nor CCT showed any statistically significant changes.

In 2014 O'Brart et al. (7) proposed a long-term observational case series study in a 20-year follow-up to evaluate PRK effects in a population of 42 patients, that underwent PRK 20 years earlier for −3 D or −6 D treatment. In this study, visual acuity, refractive error, corneal topography and AL were evaluated for each patient.

Preoperatively, B-scan ultrasonography was used to detect AL and ACD. Twenty years later, measurements were obtained using partial coherence interferometry (IOLMaster). No changes in corneal power were found between 6 months and 20 years. Eyes that had undergone a −3 or −6 correction showed increased AL and reduced ACD at 20 years, both statistically significant. As the cohort's AL increased over 20 years, authors supposed that this is likely to be the cause of myopic drift over time, rather than the regression of corneal surface correction.

In a 2020 study, De Bernardo et al. (28) evaluated ACD measurements before and after PRK by comparing IOLMaster with Pentacam. One hundred twenty-five eyes of 125 patients treated for myopia, myopic, or mixed astigmatism were evaluated. ACD measurements were obtained preoperatively and 1, 3 and 6 months postoperatively. Both devices detected ACD reduction at follow-up, confirming the data by OBrart et al. (7), suggesting the presence of anterior segment remodeling after PRK. The ACD mean difference between the two instruments was significant both in preoperative and postoperative evaluations (*p* < 0.05).

### Changes after LASIK

In a 2010 study, Chalkiadakis et al. (29) performed AL measurements using IOLMaster pre and post LASIK to treat 10 eyes of 5 patients with myopic refractive errors ranging from −2.50 to −8.00 spherical equivalent diopters (mean: −5.23 ± 1.30 D). A statistically significant AL decrease was found (*p* < 0.05).

In 2012 Wang et al. (30) evaluated ACD changes and crystalline lens thickness (LT) in 66 eyes from 66 patients (37 females and 29 males) undergoing LASIK.

Preoperative and postoperative ACD was obtained using Pentacam, Orbscan, IOLMaster and A-scan ultrasonography; AL was obtained with IOLMaster and LT was obtained with the A-scan ultrasonography.

At 1 week, 1, 3, and 6 months after LASIK, a significantly ACD decrease, measured with A-scan, IOLMaster, Orbscan and Pentacam, was seen. LT significantly increased at each follow-up evaluation (*p* < 0.001).

Compared to the preoperative values, the mean AL was found to be decreased significantly at 1 week, 1, 3, and 6 months after LASIK, with no significant changes between each post operative follow up interval.

## Discussion and conclusion

Corneal remodeling has been reported in several studies following CRS, but the idea that this remodeling does not only affect the cornea but the entire anterior segment has gradually became clear, further demonstrating that the cornea is not an inert entity but dynamically reactive to the induced changes. Moreover, ACD changes could be related to the age of patients (31), and the corneal magnification may play a role to cause a difference between pre and postoperative measurements (32). AL changes have been described after cataract surgery too (33), but in this case, the reason could be the lens extraction, which causes a decrease in the eye volume. Other unexpected changes can be detected in case of RK, where the presence of deep cuts, can induce corneal dehiscence during phacoemulsification that can change the anterior segment morphology (34, 35).

Winkler von Mohrenfels et al. (24) measured the AL pre and post LASEK with IOLMaster without detecting statistically significant difference; hypothesis, then, rejected by Rosa et al. (25) who found an error in the statistical evaluation in Winkler von Mohrenfels' results, and showed a statistically significant AL reduction after PRK. Rosa et al. provided three hypotheses to justify this finding: (1) excess of the actual ablation depth compared to the predicted one; (2) overestimation of ablation depth by the device; (3) Corneal plane posteriorly shifting. Regarding the first one, the Munnerlyn formula has been shown to underestimate the ablation depth, and it required a correcting factor to give more accurate results. However, despite the use of the correcting factor, the AL reduction does not correspond to the estimated theoretical ablation depth. About the second one, according to Rosa et al., it is widely known that there is a disagreement in the measurements of corneal thickness using various methods following refractive surgery. This could be related to the different sound or light speeds in these eyes. About the third theory, it has been shown that if the residual stromal bed is <250 μm, the laser causes corneal structural changes that result in a change in central shape adding to those caused by the treatment, so deep ablations could induce ectasia. Data from Rosa et al. (25) conclude that there is no correlation between IOLMaster measurements and the predicted ablation, supporting the idea that after CRS, the entire anterior eye segment is remodeled.

Results of Chalkiadakis et al. (29) emphasize the concept of poor correlation between the theoretical ablation depth of the excimer laser treatment and the AL variations, as measured with the IOLMaster. In fact the authors found that, on average, the ablation depth is 0.0734 mm and the AL change is, on average, 0.12 mm. Lamellae of the stromal component are permanently severed at the edges of the flap. The authors assume that the stromal disruption at the surgical ablation interface might alter the known corneal hydration gradient, that would increase from anterior to posterior (36). This latter change modifies the spacing between the lamellae and the fibers and may result in backscatter components.

A limitation of this pilot study is the small sample of patients recruited, unfortunately so far no other study to investigate this topic have been conducted. Moreover, it shows that biomechanical effects are also present in LASIK surgery, where the stromal lamellae are irreversibly damaged at the flap's borders.

Conversely, O'Brart et al. (7) found AL growth 20 years after CRS and the documented increase suggested that myopic drift over time is probably due to the continuous AL growth, rather than the regression of the correction on the corneal surface.

In fact, the authors state that, although previous studies showed that AL measured by partial coherence interferometric biometers are on average 0.15 mm longer (37) than those measured by ultrasound, this value is much lower than the mean of 0.84 mm found in their study. O' Brart et al. noted that the AL increase remained statistically significant (*p* < 0.03) even after subtracting 0.15 mm from postoperative values.

Rosa et al. (27) found no significant changes at every follow up in the AL or CCT after hyperopic PRK, unlike myopic PRK, where CCT decreased. Central corneal remodeling was hypothesized to do not sufficiently change the CCT or AL, because tissue remodeling mostly involves the mid-periphery. Therefore, it is difficult to give a clarification, based on studies in literature, on what are the changes in AL after CRS. Furthermore, from this literature review clearly emerges that CRS leads to a reduction in ACD, with each surgical technique. Demirok et al. (23) hypothesized that macro or micro corneal perforations after RK may induce a central corneal flattening and ACD reduction, but the contact measurement method was a limit of the study, because the contact of the solid tip of the probe with the cornea can cause its indentation and thus an underestimation of ACD. Rosa et al. (26) showed that, although the PRK technique involves the corneal stroma and no other ocular structure, it leads to ACD decrease, detected with two devices (IOLMaster and Orbscan II).

De Bernardo et al. (28) showed the ACD decreasing after PRK both with IOLMaster and Pentacam, giving support to the hypothesis of whole anterior segment remodeling following CRS. The corneal thinning following PRK may account for the drop in ACD, and there is strong agreement with Pentacam readings taken from the epithelium, and the IOLMaster, which measures ACD from the corneal surface to the anterior surface of the lens. Moreover, results showed a reduction in postoperative ACD even in measurements, obtained with Pentacam, from the endothelium to the lens surface, suggesting that ACD reduction is not only due to the corneal thinning. This evidence reinforces the hypothesis that PRK, removing Bowman membrane and anterior stroma, causes remodeling of the cornea. The relaxing of peripheral lamellae of corneal stroma left after PRK, may result in rearrangement of elastic forces in the corneal tissue and consequently in ACD decrease. O'Brart et al. (7) demonstrated persistence of statistically significant ACD decrease 20 years after PRK and Wang et al. (30) detected a statistically significant amount of decrease in ACD even after LASIK, along with a significant increase of the LT in all the measurements. The authors assumed that, despite the use of tropicamide and the consequent reduction in accommodative capacity, residual accommodation may contribute to the LT increase, consequently the ACD decrease after LASIK might be due to residual accommodation.

The purpose of this review, above highlighting changes after CRS, that have important implications on clinical and surgical practice, could favor new studies to bring out scientific evidence supporting the cause of the unexpected ocular morphological changes after CRS, especially the AL and ACD reduction.

## Author contributions

MD and SP analyzed the literature and wrote the original draft. NR conceived the article and reviewed the manuscript. All authors have read and approved the final manuscript.

## Conflict of interest

The authors declare that the research was conducted in the absence of any commercial or financial relationships that could be construed as a potential conflict of interest.

## Publisher's note

All claims expressed in this article are solely those of the authors and do not necessarily represent those of their affiliated organizations, or those of the publisher, the editors and the reviewers. Any product that may be evaluated in this article, or claim that may be made by its manufacturer, is not guaranteed or endorsed by the publisher.
